# The 24/7 lifestyle and male hormonal health: how sleep deprivation and ultra-processed foods impact testosterone and reproduction

**DOI:** 10.1007/s11154-026-10030-z

**Published:** 2026-03-21

**Authors:** Bianca Camilo Schimenes, Tathiana A. Alvarenga, Mariana Toricelli, Mariana Moyses-Oliveira, Matheus Brandão Vasco, Sergio Tufik, Monica Levy Andersen

**Affiliations:** 1 Departamento de Psicobiologia, Universidade Federal de São Paulo (UNIFESP), Napoleão de Barros, 925 Vila Clementino - 04024-002, São Paulo, Brazil; 2 Associação Fundo de Incentivo à Pesquisa (AFIP), São Paulo, Brazil; 3 Sleep Institute, Associação Fundo de Incentivo à Pesquisa (AFIP), São Paulo, Brazil; 4 Departamento de Cirurgia, Disciplina de Urologia, Universidade Federal de São Paulo (UNIFESP), São Paulo, Brazil

**Keywords:** Sleep, Sleep deprivation, Ultra-processed foods, Testosterone, Reproduction

## Abstract

Sleep is a fundamental physiological state essential for the regulation of multiple biological systems, including hormonal homeostasis. However, in modern society, both the duration and quality of sleep have been progressively declining. Emerging evidence suggests that sleep deprivation (SD) leads to reduced testosterone levels, which may negatively affect male reproductive health. Concurrently, the excessive consumption of ultra-processed foods (UPF) represents another major global health concern. Beyond increasing the risk of cardiometabolic disorders, UPF intake has been linked to decreased testosterone concentrations. As the primary male sex hormone, testosterone plays a critical role in maintaining numerous physiological functions, and its deficiency has been associated with a number of negative impacts including systemic inflammation, insulin resistance, increased cortisol secretion, premature aging of testosterone-producing Leydig cells, blood-testis barrier (BTB) disruption, melatonin decrease, and diet-related transcriptional remodeling of testicular cell populations. These factors may underlie the reduction in serum testosterone levels observed in both individuals with SD and with high UPF consumption. Given that SD and UPF can independently decrease testosterone levels, their potential combined impact within the context of a modern 24/7 lifestyle requires greater attention. The association between insufficient sleep and poor dietary habits may have significant implications for male reproductive health, potentially contributing to infertility and disrupting normal physiological functions. This review draws attention to the complex interplay of lifestyle factors in shaping male hormonal health, emphasizing the importance of integrative perspectives. This narrative review discusses how the combined impact of SD and UPF consumption may exert convergent or additive effects on testosterone regulation, with broad implications for reproductive function and public health strategies.

**Graphical abstract:**

**Figure:**
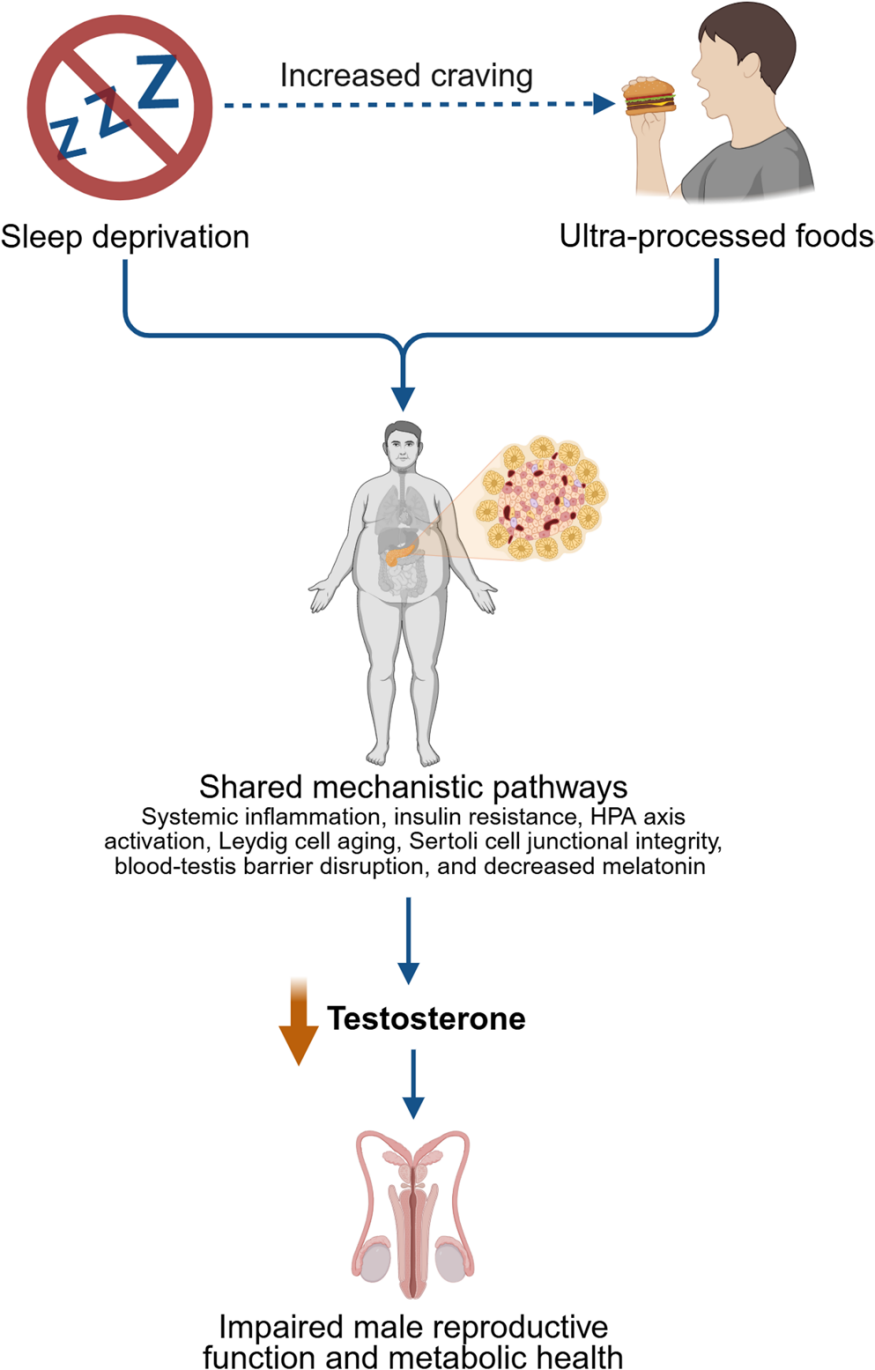
Proposed integrative model linking sleep deprivation (SD) and ultra-processed food (UPF) consumption to decreased testosterone levels, and impaired male reproductive function through common biological mechanisms

## Introduction

According to the American Urological Association (AUA), testosterone deficiency is defined as total serum testosterone levels below 300ng/dL, confirmed by at least two separate measurements. This cutoff was established based on evidence that men within this range commonly experience related symptoms, including decreased energy and endurance, fatigue, reduced physical or occupational performance, visual disturbances, anosmia, poor concentration, irritability, low motivation, depression, reduced libido, and erectile dysfunction. Total serum testosterone is commonly measured in men presenting with infertility, reflecting its recognized role in male reproductive function [1]. Testosterone concentrations are typically lower in older men, especially in those with comorbidities, with an annual reduction in total testosterone of approximately 0.4% being common [2–4]. However, additional factors, such as sleep and diet can also influence testosterone production and secretion [5–12].

Sleep is essential for regulating various biological functions, including cellular, organic, and systemic homeostasis [13–15]. It plays an important role in neuroendocrine regulation [16] and is fundamental to reproductive health in both men and women. Disruptions in sleep may contribute to impairments in reproductive function, potentially leading to infertility [17, 18].

Modern society increasingly prioritizes productivity over sleep, often underestimating its crucial role in health [19]. A cross-sectional study collected data from 9,004 adults in the United States between 2017 and 2020 and revealed that almost 30% of the participants had trouble sleeping, and 27.2% had daytime sleepiness [20]. According to the ongoing São Paulo Epidemiologic Sleep Study (EPISONO), conducted in 1987, 1995, 2007 and 2018, the incidence of sleep disorders in São Paulo (Brazil) has increased significantly over the years, especially between 1995 and 2007 [21–23]. A systematic review based on studies published between 1988 and 2023 reported that the most prevalent sleep disorders worldwide were obstructive sleep apnea (OSA) (46%), poor sleep quality (40%), insomnia (29%), and excessive daytime sleepiness (19%), while other sleep problems affected 37% of the global population [24].

Several factors contribute to sleep deprivation (SD), including high work demands, extended working hours, smoking, alcohol consumption, obesity, anxiety, depression, older age, physical activity levels, marital status, and sex [25]. Regarding the latter, studies have indicated that male individuals are more likely to sleep less than females [26–28].

Although often underestimated, lack of sleep has become a public health concern. A review highlighted that insufficient sleep duration can lead to cognitive impairment, cardiovascular disease, decreased immune response, obesity, diabetes, burnout, certain cancers, an increased risk of premature mortality, and daytime sleepiness, which raises the risk of accidents [25]. Moreover, SD has been linked to reduced testosterone levels in men, potentially compromising male reproductive health [5–7, 29].

In parallel, the modern diet, particularly the high intake of ultra-processed foods (UPF), represents one component of contemporary lifestyle factors influencing global population health [30], including hormonal balance, metabolic regulation, and testicular function.

Testosterone, the primary male hormone, is produced in Leydig cells in the testes in response to luteinizing hormone (LH), which is stimulated by the hypothalamic gonadotropin-releasing hormone (GnRH). This androgen participates in the development of secondary sex characteristics, promoting voice change, muscle and body hair development, sexual desire, spermatogenesis, maintenance of muscle mass and strength, hematopoiesis, visceral fat reduction, and cognitive function [31].

Sleep and diet are both recognized as global health concerns in contemporary society, including in respect of their potential influence on testosterone levels. Understanding the interplay between these two factors is essential to assess their potential joint influence on male reproductive health and quality of life. In this narrative integrative review, we discuss the effects of SD and UPF intake on endocrine regulation, with a particular focus on testosterone levels. We will also explore the underlying molecular mechanisms, highlighting the need for an integrated and up-to-date perspective on this topic.

## Search strategy and study selection

This review was conducted as a narrative integrative synthesis aimed at conceptually linking SD, UPF consumption, and testosterone regulation.

The literature search was performed in PubMed up to December 2025. The following key terms and their combinations were used: *sleep*,* sleep deprivation*, *sleep restriction*, *sleep disorders*, *ultra-processed foods*, *diet*, *dietary habits*,* dietary patterns*,* testosterone*, *male reproductive health*, *reproduction*,* Leydig cells*, *inflammation*, and *insulin resistance*. Boolean operators (“AND”, “OR”) were applied to refine searches.

We included original experimental and observational studies, reviews, and mechanistic reports written in English that addressed the relationship between sleep, diet, metabolic regulation, and male reproductive hormones. Exclusion criteria comprised studies not involving male subjects or male animal models, articles not addressing endocrine or reproductive outcomes, conference abstracts, and non-peer-reviewed literature.

Titles and abstracts were screened for relevance, followed by full-text evaluation when appropriate. Studies were selected based on their conceptual contribution to the proposed integrative framework rather than formal risk-of-bias scoring, which is consistent with the narrative nature of the review.

## The role of sleep and sleep deprivation in male hormonal regulation

Testosterone secretion is closely linked to sleep and follows a circadian rhythm, with levels peaking in the morning and gradually declining throughout the day [32, 33]. The discharge of LH and testosterone occurs in constant pulses [34]. In younger men, LH pulses are less frequent during the night and follow a well-defined circadian pattern, whereas in older men, this rhythm is markedly attenuated [35].

The disruption of hormonal regulation by SD can pose a significant threat to reproductive health. A study from our group demonstrated that male Wistar rats subjected to SD had significantly lower testosterone alongside elevated progesterone concentrations [5]. Similar results were found in another study, with testosterone levels not returning to control levels even after a recovery period equivalent to the time spent in SD, although progesterone levels did [6]. Adult male rats exposed to paradoxical sleep deprivation (PSD) not only showed a significant decrease in testosterone concentrations, but also impairments in sexual motivation and performance [36]. Interestingly, when PSD was combined with food restriction, these negative effects on sexual behavior were no longer observed [37]. SD can lead to a reduction in testosterone concentration regardless of its duration. It has been suggested that the decrease of this hormone in sleep-deprived male rats may be due to the inhibition of testosterone production by increased levels of serotonin, and reduced expression of testicular steroid acute regulatory protein [7].

Experimental evidence also implicates central regulation of the hypothalamus-pituitary-gonadal (HPG) axis in SD-induced testosterone suppression. In a rat model of 72-hour total SD, Lee et al. demonstrated marked reductions in LH and subsequent testosterone levels, without significant changes in hypothalamic GnRH expression, suggesting that SD can induce secondary hypogonadism via pituitary modulation of gonadotropin release. This central suppression of LH was associated with downstream effects on erectile tissue, including alterations in nitric oxide synthase signaling, further linking HPG axis disruption to functional reproductive outcomes [38].

While animal studies have consistently documented a decline in testosterone following SD or sleep restriction, recent human evidence provides a more nuanced picture of this relationship. A systematic review and meta-analysis of controlled studies showed that total SD (≥ 24 h) significantly reduces serum testosterone levels in healthy men, whereas short-term partial sleep restriction did not have a significant effect on testosterone concentrations [12]. In addition, data from the National Health and Nutrition Examination Survey (NHANES) 2011–2016 cohort indicate that the association between habitual sleep duration and testosterone levels varies by age group in men; for example, short sleep (≤ 6 h) was associated with higher testosterone in younger adults (20–40 years), while in middle-aged men (41–64 years) longer sleep duration was linked to lower testosterone [39]. These findings highlight that both acute and chronic patterns of sleep may influence male endocrine regulation, although the direction and magnitude of this association differ across experimental and population settings.

Research indicates that social jet lag, a misalignment between biological and social sleep schedules, tends to be less pronounced in girls than boys. Adolescent boys exhibit longer sleep onset latency at weekends and less REM sleep during weekdays compared to girls [40]. These observations raise the hypothesis that social jet lag may contribute to clinically relevant alterations in the male adolescent reproductive system [41]. Consistent with this, a systematic review by Caetano et al. described that short sleep duration and evening chronotype may contribute to infertility. Men with shorter sleep duration were more likely to experience sexual dysfunction and reduced sexual activity, while those with an evening chronotype presented a decreased sperm count and reduced motility [42]. A meta-analysis within this study observed a strong association between sleep disturbances and a lower sperm count, reduced sperm concentration, and a decrease in normal morphology [43]. It is important to note that the review had limitations related to heterogeneity in the study methodologies and a lack of objective sleep measurements, underscoring the need for more research investigating the relationship between sleep and reproduction.

Androgen deficiency is related to a range of health disorders, including insulin resistance, metabolic syndrome, mood disturbances, loss of muscle mass, increased visceral fat, anemia, and decreased bone mineral density [44–50]. Other symptoms, such as reduced libido, cognitive impairment, low energy, and sleepiness, are commonly associated with both low testosterone levels and SD [51, 52]. There is considerable similarity between the symptoms associated with SD and high consumption of UPF, many of which are linked to testosterone deficiency [25, 53–55]. This overlap makes it plausible to suggest that poor sleep and a UPF-rich diet could converge through shared pathways, potentially influencing hormonal health, particularly testosterone regulation.

Overall, the association between sleep duration and testosterone is supported by controlled laboratory studies showing acute reductions in testosterone following total SD, providing moderate causal evidence for short-term effects. However, most long-term and population-based data remain observational, and studies report attenuation or even directionally different associations after adjustment for adiposity, age, or comorbidities. Moreover, partial sleep restriction does not consistently reduce testosterone in experimental settings. While short-term experimental evidence supports a causal role of severe sleep loss on testosterone suppression, the magnitude, direction, and persistence of this effect in real-world conditions remain uncertain.

## Ultra-processed food: an emerging endocrine disrupting agent

UPF are widely consumed due to their affordability and long shelf life [56], yet they are consistently associated with increased metabolic risk and hormonal dysregulation [55, 57]. Certain additives, such as phthalates and bisphenol A (BPA), may disrupt androgen receptor signaling and inhibit key enzymes involved in testosterone biosynthesis [55].

A global investigation into UPF intake revealed that the United States and the United Kingdom had the highest levels of total energy intake derived from UPF, ranging between 50% and 70%. In contrast, Italy reported the lowest consumption level, with UPF contributing to about 10% of total energy intake [30]. This disparity may be explained by the widespread adherence to the Mediterranean diet in countries located in the Mediterranean region, a dietary pattern rich in fruits, vegetables, whole grains, dairy products, and unsaturated fats. Unlike UPF, the Mediterranean diet is associated with protective effects against several chronic conditions, including type 2 diabetes mellitus (T2DM), cancer, cardiovascular and neurodegenerative diseases, as well as reduced overall mortality [58].

It is well known that diet directly impacts the functioning of physiological systems. When not well balanced, it can have significant negative consequences for overall health. Evidence indicates associations between UPF consumption and an increased risk of developing several metabolic disorders, including obesity, T2DM, cardiovascular diseases, cancer, increased risk of depression, and endocrine disruption [53–55, 57, 59, 60].

Beyond cardiometabolic effects, UPF intake may also interact with central neuroendocrine regulation. Metabolic hormones, including leptin, insulin, and ghrelin, which are altered in the context of poor diet quality [61–63], modulate hypothalamic control of energy balance and GnRH secretion, linking eating circuits and reproductive signaling [64]. Both sleep loss and metabolic stress alter HPA axis activity and appetite hormone balance [65, 66], suggesting a hormonal-metabolic crosstalk that connects diet, stress physiology, and reproductive endocrine control.

Studies have identified a direct link between dietary patterns and testosterone levels [8–10]. Romero et al. (2012) reported a significant decline in both total and free testosterone levels in male and female rats fed a cafeteria diet, a UPF-rich regimen adapted for research animals [67], compared to its controls, with the most pronounced decrease observed in free testosterone among males (−68%) [8]. More recently, Ceretti et al. (2024) extended these findings to humans, showing that high consumption of UPF was associated with lower sperm concentration and motility in healthy young men, suggesting a detrimental impact of UPF intake on male reproductive parameters [68].

A cross-sectional study conducted in Taiwan among middle-aged and elderly men with impaired kidney function found that a fried-processed dietary pattern, characterized by high intake of calorie-dense and industrially processed foods, was associated with lower serum testosterone levels and a reduced testosterone-to-triglyceride ratio compared with healthier dietary patterns [9]. In a separate cross-sectional analysis of men of reproductive age, higher consumption of UPF was inversely associated with total sperm count, sperm concentration, and total sperm motility, suggesting a negative relationship between UPF intake and key semen quality parameters [69]. Consistent with these findings, a study of healthy young men in Italy reported that those consuming the highest amounts of UPF had significantly lower sperm concentration and reduced progressive motility compared with men with lower UPF intake [68]. Similar results were found among younger Taiwanese males aged 20 to 64 years old, who had unhealthy dietary patterns associated with low serum total testosterone levels and hypogonadism [10].

Food additives commonly found in UPF, used to enhance taste, texture, appearance, and the shelf life of processed foods, have been identified as endocrine-disrupting chemicals. These include BPA, tartrazine, phthalates, erythrosine, parabens, and artificial sweeteners. These additives can interfere with the normal function of the endocrine system, increasing the risk of metabolic disorders and impairing reproductive health through various mechanisms, including androgen synthesis, receptor binding, and signaling (Table 1) [55].

**Table 1:** Overview of key toxicants commonly associated with ultra-processed food environments and packaging, their molecular initiating events, and downstream testicular endpoints related to testosterone regulation and spermatogenic dysfunction

UPF-associated toxicant	Molecular initiating events	Measurable testicular endpoints
Acrylamide [70]	Induction of oxidative stress; mitochondrial dysfunction; down-regulation of steroidogenic enzymes (e.g., StAR, CYP11A1, 3β-HSD)	• Degeneration of spermatogenic cells • Imbalance of testicular biomarkers (malondialdehyde, nitric oxide, superoxide dismutase, catalase, and glutathione) • Impaired spermatogenesis • ↓ Sperm quality • ↓ Testosterone levels
Advanced glycation end-products (AGEs) [71]	Accumulation of AGEs in reproductive tissues and activation of the AGE receptor (RAGE); increased oxidative stress, inflammation, and disruption of testicular antioxidant defenses	• ↓ Sperm count and motility • ↑ Sperm morphological abnormalities • ↑ Sperm DNA fragmentation • ↓ Testicular antioxidant capacity
Artificial sweeteners (aspartame) [72]	Oxidative stress generation; decreased LH/FSH secretion; mitochondrial dysfunction in Leydig cells	• ↓ Serum testosterone • ↓ Serum LH and FSH • Reduced sperm quality • Testicular cells apoptosis
Micro-plastics [73]	Induction of oxidative stress inflammation; reduction in seminiferous tubule diameter; impaired GnRH secretion; reduced testosterone synthesis	• ↓ Leydig cell number • ↓ Testosterone levels • Disrupted spermatogenesis • Abnormal sperm morphology and motility

Recent experimental evidence from controlled human feeding studies suggests that UPF may exert biologically disruptive effects beyond poor nutrient quality per se. A controlled crossover trial has shown that diets high in UPF, even when matched for calories and macronutrients with minimally processed diets, lead to increased adiposity, perturbations in cardiometabolic biomarkers, and declines in reproductive hormones including testosterone and follicle-stimulating hormone (FSH), implicating both metabolic and endocrine pathways. These effects have been associated with increased exposure to phthalates and shifts in hormonal balance, supporting the notion that industrial processing and associated additives may contribute to endocrine disruption in addition to the effects of diet composition and energy balance [74].

It is important to note that diet can affect not only testosterone levels, but also male sexual behavior. Our group previously demonstrated that rats fed a high-fat cafeteria diet for nine weeks, being submitted to SD afterwards and injected with cocaine, had a significant decrease in erection when compared to those receiving standard diet independently of SD and cocaine interventions [5]. Studies have indicated that SD is also associated with alterations in eating behavior, as it can disrupt the regulation of appetite related hormones, such as leptin and ghrelin, leading to increased appetite and a greater preference for high-calorie foods [16, 75]. Thus, SD may contribute to the reduction of testosterone levels not only directly, but also potentially indirectly through changes in dietary behavior. These findings suggest that high UPF intake may negatively impact male reproductive health, which could be further influenced by coexisting SD. Given that both factors are increasingly common in modern lifestyles, their combined effects are a growing concern.

Evidence linking UPF consumption to testosterone and male reproductive parameters remains largely indirect. Few experimental animal model studies associate ultra-processed diets with testosterone levels. In humans, most available data derives from cross-sectional or cohort studies assessing UPF intake in relation to testosterone levels or semen quality, which demonstrate associations rather than causality. While some studies report inverse associations between UPF consumption and testosterone concentrations, most report effects primarily on sperm parameters rather than on circulating hormone levels. Despite strong biological plausibility, direct causal evidence connecting UPF intake to testosterone suppression and impaired male reproduction in humans remains limited and partially inconsistent.

## The bidirectional circadian–testis axis linking ultra-processed foods to male reproductive endocrinology

Recent evidence indicates that diet quality participates in circadian regulation relevant to male reproduction. Higher consumption of UPF is associated with poorer sleep quality, greater insomnia risk, and altered sleep timing in population studies, suggesting disruption of behavioral circadian rhythms [76, 77]. Experimental and clinical data further show that circadian misalignment and sleep loss blunt nocturnal melatonin secretion, a key signal coordinating peripheral clocks, including those in the testis [78–80]. In parallel, a Mendelian-randomization analysis demonstrated that genetically determined chronotype is causally associated with morning testosterone levels in men [81], while actigraphy-based cohorts report that delayed sleep midpoint and social jet lag are linked to lower sperm concentration and total sperm count [82]. Integrating these findings, UPF-related circadian disruption may potentially attenuate melatonin signaling and desynchronize testicular steroidogenic rhythms, suggesting a possible feed-forward interaction between impaired sleep timing and reduced testosterone availability.

### Intervention gaps and circadian-realignment strategies

Few randomized trials have examined whether lifestyle or dietary interventions can restore testosterone or semen parameters through mechanisms potentially involving circadian alignment. In healthy young men, a six-month Mediterranean diet intervention increased sperm concentration and total sperm count compared with a low-fat diet, suggesting diet quality improvements may positively affect spermatogenesis [83]. In infertile men, a randomized clinical trial showed that both Persian Medicine diet and Mediterranean diet interventions improved erectile function, semen count, motility, and morphology [84]. A controlled dietary modification study reported that a low-carbohydrate Mediterranean-style diet was associated with increased testosterone and decreased sperm DNA fragmentation after three months, indicating that specific macronutrient profiles may influence both hormonal parameters and semen integrity [85]. Evidence on melatonin supplementation directly affecting male reproductive hormones in humans is limited and mixed; a small crossover study found no consistent changes in semen quality or hormone levels with melatonin treatment, underscoring the need for larger, mechanistic trials [86].

Overall, while Mediterranean dietary patterns appear promising for improving semen parameters, the extent to which benefits are mediated by weight loss, inflammatory attenuation, or direct testicular effects, including through circadian realignment or melatonin pathways, it warrants future targeted randomized intervention research.

## The interplay between sleep and diet - its effects on male reproductive health and potential mechanisms

Multiple biological pathways may mediate the hypothesized convergence of SD and UPF consumption on hormonal regulation and male reproductive health. As discussed, both SD and the consumption of UPF have been associated with decreased testosterone levels. However, the underlying mechanisms responsible for this effect remain unclear. Regarding molecular mechanisms, there are shared biological pathways between SD, diet and male reproduction.

Our research group has previously explored the shared genetic basis of erectile dysfunction (ED) and OSA through in silico analysis, identifying thirty-five common genes between the two conditions. Among these thirty-five shared genes, functional enrichment analysis pointed to biological processes related to lipid metabolism, a molecular pathway frequently disturbed in the context of UPF consumption [87, 88]. In another study, we investigated the genetic link between male factor infertility and insomnia, identifying twenty-eight shared genes and an enrichment of kinesin-binding pathways among these overlapping genes. Protein-protein interaction analysis highlighted kinesin-related proteins PLEKHM2 and KCL1, suggesting that disruptions in kinesin-related pathways may connect these conditions [89]. Interestingly, this same pathway has been linked to metabolic dysfunction-associated fatty liver disease (MALFD). Miao et al. explored the role of Kif13b, a motor protein, in mouse models with MAFLD and observed a reduction in Kif13b gene expression in animals with this condition [90]. It was found that Kif13b deficiency led to hepatic steatosis and worsened diet-induced steatohepatitis. Mechanistically, Kif13b depletion increased lipid synthesis and impaired mitochondrial oxidative phosphorylation [90]. These findings indicate that further exploration of the interplay between male infertility, insomnia, and metabolic dysfunctions might consider kinesin-related pathways.

**Fig. 1:**
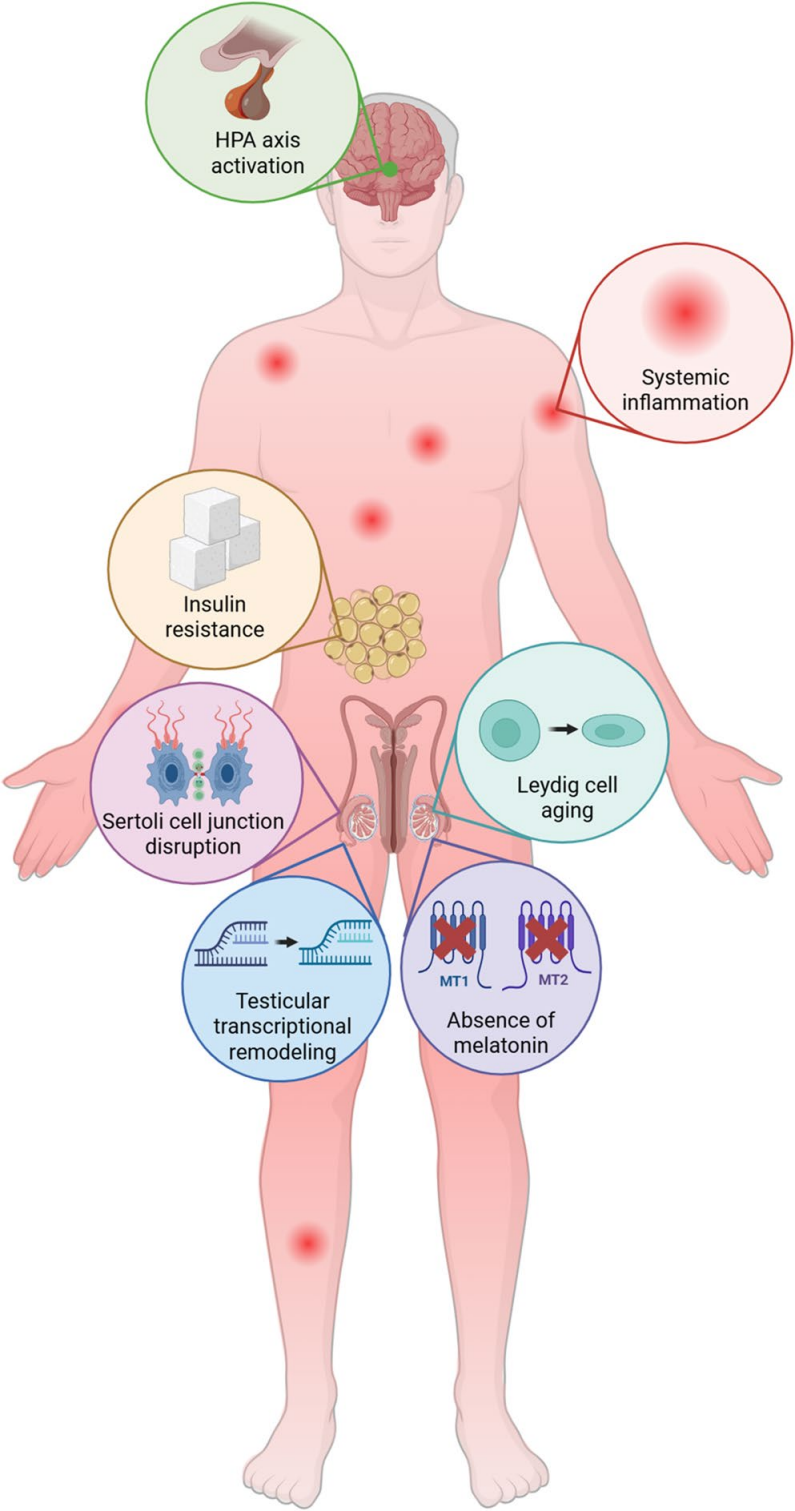
Schematic illustration of the mechanisms linking sleep deprivation (SD) and ultra-processed food (UPF) consumption to testosterone dysregulation and male reproductive impairment

**Fig. 2:**
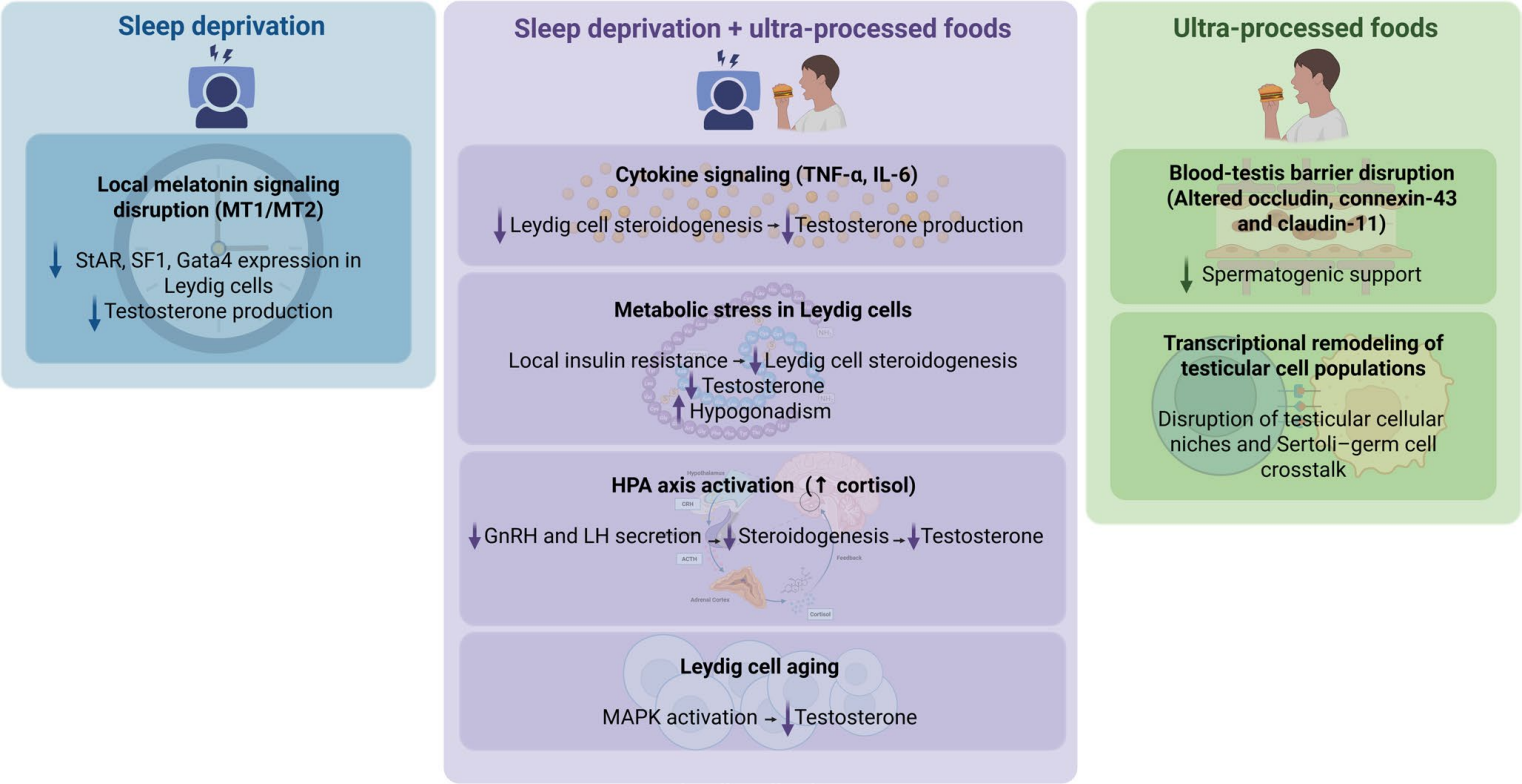
Individual and shared mechanisms by which sleep deprivation (SD) and ultra-processed food (UPF) consumption may impair testosterone regulation and male reproductive function

At present, no studies directly tested the combined exposure to SD and high UPF intake on testosterone or male reproductive outcomes. The proposed interaction is therefore conceptual and based on the convergence of shared pathways, including systemic inflammation, insulin resistance, HPA axis activation, Leydig cell aging, Sertoli cell junctional integrity, melatonin production, and diet-related transcriptional remodeling of testicular cell populations (Figs. 1 and 2). While each factor independently shows associations with hormonal regulation, their joint effects remain speculative.

### Systemic inflammation

SD has been consistently linked with elevated systemic inflammation. In experimental models, male mice exposed to SD showed increased levels of inflammatory markers, in part due to compromised gut barrier integrity, which may facilitate the translocation of microbial components such as lipopolysaccharides (LPS) into the circulation. The increase of circulating LPS triggered the activation of pro-inflammatory pathways, including tumor necrosis factor alpha (TNF-α) [57], ultimately impairing Leydig cell function and testosterone production. The inflammatory response observed under SD conditions has been related to gut dysbiosis. Germ-free mice that received microbiota transplants from SD-exposed donors exhibited activation of the Toll-like receptor 4 (TLR4)/nuclear factor κB (NF-κB) inflammatory pathway, supporting a causal role of the gut microbiota in SD-induced inflammatory responses in rodents [91].

Similarly, in humans, the evidence is more indirect. A probiotic intervention improved sleep quality by 69%, both in the general population and in elite athlete groups. These improvements were associated with a reduction in the oxidative stress marker derivatives of reactive oxygen metabolites (D-ROMS) and an elevated free testosterone/cortisol ratio [92]. Resident physicians subjected to SD exhibited higher levels of C-reactive protein (CRP), indicating the onset of low-grade systemic inflammation [53]. However, these studies do not establish direct causality between SD, inflammation and testosterone regulation in humans.

The consumption of UPF has been strongly associated with the development of several non-communicable diseases, often mediated by the onset of systemic inflammation [93, 94]. This inflammatory state is mainly driven by the increased adiposity and obesity caused by UPF intake, which are recognized as chronic, low-grade inflammatory conditions [95]. A cross-sectional study investigated the relationship between diet quality and inflammatory markers in middle- and older-aged men and women. Elevated levels of pro-inflammatory cytokines, including interleukin 6 (IL-6), and TNF-α, were found to be related to higher UPF consumption [93]. Recently, Urrutia-Pereira et al. highlighted the role of food additives present in UPF in the development of inflammation through the disruption of the intestinal barrier [94].

Regarding testosterone regulation, inflammation mediated by LPS intraperitoneal injection in male mice led to impaired reproductive function, characterized by lower testosterone levels, downregulation of testicular gene expression involved in steroidogenesis, decreased spermatogenesis, and higher levels of LH, suggesting a disruption of the HPG axis [96]. In humans, evidence is largely associative: in elderly men, elevated levels of the inflammatory marker high-sensitivity C-reactive protein (hs-CRP) were associated with lower serum total testosterone levels [97]. A high systemic immune-inflammation index (SII), calculated by neutrophil-to-lymphocyte count ratio, was linked to testosterone deficiency in young adult men [98].

Collectively, these findings position inflammation as an active regulator of testicular function, linking sleep- and diet-induced inflammatory activation to disruption of the HPG axis, and reduced intratesticular and circulating testosterone levels, rather than merely reflecting a systemic comorbidity.

### Insulin resistance

Insulin resistance is a condition commonly associated with systemic inflammation, obesity, and poor sleep [99, 100]. Evidence has revealed that chronic SD may potentially induce insulin resistance and impact testosterone levels in men [99, 101, 102]. Similarly, UPF intake can induce insulin resistance and significantly increase the risk of developing T2DM [103, 104].

A study by Pitteloud et al. reported that men who had impaired glucose tolerance and T2DM had lower levels of testosterone compared to those with normal glucose tolerance. They noted that the decrease in testosterone levels associated with insulin resistance was not related to the HPG axis, demonstrated by normal endogenous LH secretion and LH response to GnRH [105]. It was hypothesized that the mechanism underlying the effect of insulin resistance on testosterone levels may involve impaired steroidogenesis in Leydig cells, possibly due to a local resistance to insulin action, which plays an important role in stimulating testosterone production in these cells [105, 106]. Grossmann et al. documented an inverse association between testosterone levels and insulin resistance in men with type 1 and type 2 diabetes [101]. Patients with hypogonadism exhibited higher insulin levels and Homeostasis Model Assessment of Insulin Resistance (HOMA-IR) scores, both of which are indicators of insulin resistance [107]. Another study identified a strong link between total serum testosterone and insulin resistance, measured by logHOMA-IR [108].

Together, these findings indicate that insulin resistance may act as a local modulator of Leydig cell sensitivity and testosterone biosynthetic capacity.

### Cortisol and HPA axis activation

Emerging evidence suggests that UPF consumption may influence stress physiology via activation of the hypothalamic-pituitary-adrenal (HPA) axis. In a large cohort study, moderate UPF intake was associated with elevated hair cortisol levels, a biomarker of chronic stress, even after adjustment for sociodemographic and behavioral confounders, indicating a link between diet and prolonged HPA axis activation [109].

Concurrently, SD and poor sleep quality are well-established stressors that activate the HPA axis, leading to higher baseline cortisol release and an exaggerated cortisol response to acute stress, consistent with broader dysregulation of HPA activity under sleep loss [110, 111].

Elevated cortisol has well-documented suppressive effects on the HPG axis, including inhibition of GnRH and LH secretion and direct impairment of testicular steroidogenesis, which can reduce testosterone production [112, 113].

The overlap between diet-related cortisol elevation and SD-induced HPA activation suggests an additional endocrine pathway by which modern lifestyle factors may converge to influence hormonal regulation, beyond metabolic and inflammatory mechanisms. This convergence positions HPA–HPG crosstalk as a mechanistic bridge through which sleep loss and UPF-related stress signaling directly constrain testosterone synthesis.

### Leydig cell aging

Another putative mechanism that could be involved in the relationship between sleep, diet and decreased testosterone levels is the aging of Leydig cells. While natural aging contributes to reduced testosterone synthesis, Leydig cells’ premature aging can also impair the production of this hormone.

Luo et al. demonstrated that obesity induced by a high-fat diet (HFD) in mice led to the premature aging of Leydig cells and, consequently, a decline in serum testosterone levels [114]. The molecular pathways involved were investigated, revealing the activation of p38 mitogen-activated protein kinase (MAPK) in aged Leydig cells, a protein often activated under oxidative stress and inflammatory conditions [114].

SD has been related to a significant decline in the number of Leydig cells, although testosterone levels were not analyzed in this particular study [115].

It is noteworthy that evidence linking UPF consumption or SD to Leydig cell functioning and testosterone production remains scarce in the current literature. Most of them derive from animal models, and direct validation in humans is still limited.

Taken together, these findings suggest putative mechanisms through which SD and poor diet quality may contribute to the development of testosterone deficiency, although further research is needed to clarify these associations.

### Sertoli cell junctional integrity and blood–testis barrier disruption

Emerging evidence highlights testis-centric mechanisms through which sleep disruption and UPF consumption may directly impair male reproductive function.

One important target of endocrine-disrupting chemicals associated with UPF environments is the Sertoli cell junctional network, which forms the blood–testis barrier (BTB) and preserves immune privilege. Experimental studies show that exposure to BPA and phthalate metabolites, additives commonly found in UPF, disrupts BTB integrity by altering key junctional proteins. In human Sertoli cells, BPA and monobutyl phthalate reduce the expression of occludin, zonula occludens-1 (ZO-1), N-cadherin, β-catenin and androgen receptor, indicating impairment of tight and adherens junction complexes [116]. In parallel, animal and in vitro models report decreased occludin and altered connexin-43 and claudin-11 after bisphenol exposure, supporting compromised Sertoli–germ cell communication and barrier function [117, 118].

Together, these junctional alterations may weaken BTB structure, impair Sertoli–germ cell communication, and compromise the microenvironment required for efficient spermatogenesis and androgen-dependent support of germ cell development.

### Melatonin

The testis possesses a local circadian system, with melatonin receptors MT1 and MT2 expressed in Leydig and Sertoli cells in mammalian testes, where receptor abundance correlates with steroidogenic and spermatogenic activity [119].

Experimental evidence indicates that melatonin signaling modulates steroidogenic gene expression including Steroidogenic Acute Regulatory Protein (StAR), Steroidogenic factor 1 (SF1), and Transcription factor GATA-4 (Gata4) in Leydig cells, enhancing testosterone synthesis [120]. Knockdown of melatonin receptors significantly reduces testosterone production, further supporting a local role for melatonin in testicular steroidogenesis [121].

Thus, reduced melatonin availability induced by sleep disruption or lifestyle factors may uncouple local testicular steroidogenic rhythms, ultimately impairing both the timing and amount of testosterone production [80], connecting circadian disruption with impaired Leydig cell steroidogenic capacity.

### Diet-related transcriptional remodeling of testicular cell populations

Recent single-cell RNA sequencing of testes from mice fed a HFD revealed transcriptional alterations across spermatogenic and somatic cell populations compared to lean controls. This study identified distinct clusters of spermatogonia and demonstrated that HFD alters gene expression profiles in undifferentiated and differentiated spermatogenic cells, suggesting disruptions in cellular niches and intercellular communication within the testicular microenvironment [122].

Although still emerging, these data suggest that dietary imbalance could reshape transcriptional programs and cellular niches within the testicular microenvironment, potentially affecting germline maintenance and somatic–germ cell communication networks that coordinate spermatogenesis with Leydig cell steroidogenesis and local testosterone supply. Mechanistic single-cell studies directly linking nutritional exposures to functional testicular outcomes remain needed.

## Discussion

Sleep plays a crucial role in the maintenance of general health. However, contemporary society faces serious sleep-related issues, largely attributed to increasing work demands and the accelerated pace of life, particularly in major metropolitan areas. Simultaneously, diet quality is being neglected, driven by the rise in the production of UPF by the food industry and their growing preference among the worldwide population [123, 124]. Persistent advertising, the high palatability, and the convenience offered by these products help explain, at least in part, the rise in their consumption.

It is noteworthy that men are generally more prone to shorter sleep duration compared to women [26–28], a factor that may adversely affect testosterone production and the maintenance of reproductive health. A study conducted in a university student population showed that men tend to consume greater amounts of UPF than women, particularly alcoholic beverages, energy drinks, and cereal bars [125]. It is well established that disruptions in both sleep and diet can cause deleterious effects on male reproduction, primarily by reducing testosterone levels through different biological pathways [5, 6, 8, 9, 12, 36, 39, 126–128].

The modern lifestyle, marked by chronic SD and high consumption of UPF, represents part of a cluster of unhealthy lifestyle habits that may collectively contribute to hormonal disturbances in men. The effect of these factors together may differ from their individual impacts, potentially contributing to more severe endocrine dysfunction. Sleep and diet may converge along shared pathways that influence hormonal regulation, forming a network of inflammation, insulin resistance, cellular dysfunction, circadian misalignment, and testosterone deficiency. This interaction suggests that metabolic and hormonal disturbances do not occur in isolation, but rather as part of a broader lifestyle-driven physiological imbalance.

The metabolic cost of modern lifestyles may accelerate androgenic decline, potentially leading to early-onset hypogonadism, hormonal dysregulation, and reduced sexual behavior. Studies specifically exploring the relationship between UPF intake and testosterone production and secretion remain scarce, and no studies have yet investigated the combined effects of SD and UPF consumption on testosterone levels.

Insufficient sleep may promote cravings for energy-dense UPF, whereas diets rich in UPF can further compromise sleep quality, thereby intensifying hormonal and reproductive disturbances [77]. The relationships between sleep disruption, diet quality, metabolic dysfunction, and testosterone regulation are likely dynamic and interdependent rather than linear. Reduced testosterone may impair sleep architecture and circadian regulation [129], while metabolic disease can precede and promote both sleep disturbance [130] and maladaptive dietary patterns [131]. These feedback loops limit causal inference in observational studies and support a framework in which lifestyle exposures and endocrine alterations interact over time instead of operating through isolated linear pathways.

Although growing evidence links both sleep disturbance and poor diet quality to testosterone regulation, the strength of evidence differs substantially across domains. Experimental SD studies provide moderate causal support for short-term testosterone suppression, whereas most nutritional evidence remains observational. Human data directly connecting UPF intake to testosterone or semen parameters are limited and sometimes inconsistent, and randomized dietary trials rarely include reproductive endpoints. The proposed interaction remains conceptual and based on shared biological pathways rather than direct demonstration. These limitations highlight the need for longitudinal and interventional designs that disentangle causality, temporality, and potential additive versus merely correlated lifestyle effects.

### Future perspectives

Emerging insights into men’s reproductive health highlight a possible interaction between sleep and nutrition, two fields with a similar influence on metabolic and hormonal outcomes, yet they have been largely studied separately in relation to testosterone regulation, and there is limited research in the current literature that considers the potential combined impact of these factors. This may be due mostly to the methodological challenges involved in studying dynamic, interdependent behaviors such as sleep patterns and diet quality alongside endocrine and metabolic endpoints.

Despite these challenges, it is essential that future investigations examine the joint and potentially modifying effects of sleep disturbances and high UPF intake on male reproductive health. A key starting point could be assessing whether poor sleep quality and duration, together with unhealthy dietary patterns, exert additive, convergent, or interactive influences on testosterone regulation and spermatogenic support. Future research should aim to define specific exposure thresholds, vulnerable phenotypes, and mechanistic pathways linking sleep–diet interactions to endocrine and reproductive outcomes. These advances may also inform clinical practice by reinforcing the relevance of systematic assessment of sleep and dietary habits as components of risk stratification and early endocrine dysfunction detection.

Another limitation in the current evidence is the inability to unravel whether observed associations between UPF intake and testosterone suppression are driven by the macronutrient composition of the foods themselves or by endocrine-disrupting additives they contain. While both pathways are biologically plausible, human studies are mostly observational and do not allow the relative contribution of dietary composition versus chemical exposure to be determined. Future mechanistic research is needed to clarify whether these effects are primarily nutritional, chemical, or a combination of both.

Future studies should prioritize prospective longitudinal designs and experimental approaches, integrating objective sleep assessments (e.g., actigraphy, polysomnography, circadian phase markers) with quantitative and qualitative dietary characterization capable of capturing UPF exposure and inflammatory potential (e.g., NOVA Food Frequency Questionnaire). Confounding and mediating variables, including physical activity, stress levels, adiposity and alcohol consumption should be explicitly modeled to resolve causal structure and temporal relationships.

## Conclusions

Both SD and UPF consumption are associated with alterations in testosterone regulation in men. Interestingly, the molecular pathways involved in testosterone reduction are commonly observed as consequences of SD and UPF intake. These shared mechanisms underscore the importance of investigating not only the isolated effects of sleep and diet on testosterone concentrations, but also their potential convergent or additive impact on male endocrine function.

Overall, the evidence supports a biologically plausible framework in which lifestyle-related factors reshape testosterone regulation as part of broader metabolic and reproductive disturbances, rather than acting through a single dominant pathway. Consideration of sleep patterns and diet quality in clinical practice may aid in the early recognition of endocrine alterations and support more targeted preventive strategies.

## Data Availability

Not applicable. No new data generated.

## Abbreviations

AUA: American Urological Association
BTB: Blood-testis barrier
BPA: Bisphenol A
CRP: C-reactive protein
D-ROMS: Reactive oxygen metabolites
ED: Erectile dysfunction
EPISONO: São Paulo Epidemiologic Sleep Study
FSH: Follicle-stimulating hormone
GATA4: GATA binding protein 4
GnRH: Gonadotropin-releasing hormone
HFD: High-fat diet
HPA: Hypothalamic-pituitary-adrenal
HPG: Hypothalamus-pituitary-gonadal
HOMA-IR: Homeostasis Model Assessment of Insulin Resistance
hs-CRP: High-sensitivity C-reactive protein
IL-6: Interleukin 6
LH: Luteinizing hormone
LPS: Lipopolysaccharides
MALFD: Metabolic dysfunction-associated fatty liver disease
MAPK: Mitogen-activated protein kinase
MT1: Melatonin Receptor 1
MT2: Melatonin Receptor 2
NF-κB: Nuclear factor κB
NHANES: National Health and Nutrition Examination Survey
OSA: Obstructive sleep apnea
PSD: Paradoxical sleep deprivation
SD: Sleep deprivation
SF1: Steroidogenic factor 1
SII: Systemic immune-inflammation index
StAR: Steroidogenic Acute Regulatory Protein
T2DM: Type 2 diabetes mellitus
TLR4: Toll-like receptor 4
TNF: α-Tumor necrosis factor alpha
UPF: Ultra-processed food
ZO: 1-Zonula occludens-1
